# Views of Own Body Weight and the Perceived Risks of Developing Obesity and NCDs in South African Adults

**DOI:** 10.3390/ijerph182111265

**Published:** 2021-10-27

**Authors:** Mashudu Manafe, Paul K. Chelule, Sphiwe Madiba

**Affiliations:** 1 Department of Human Nutrition & Dietetics, Sefako Makgatho Health Sciences University, Pretoria 0001, South Africa; 2 Department of Public Health, Sefako Makgatho Health Sciences University, Pretoria 0001, South Africa; paul.chelule@smu.ac.za (P.K.C.); sphiwe.madiba@smu.ac.za (S.M.)

**Keywords:** perception, overweight, obesity, non-communicable diseases, body mass index

## Abstract

Obesity has become a serious public health problem worldwide and is linked to an increased risk of non-communicable diseases (NCDs). Poor self-perception of body weight is postulated to play a negative role in curbing increased rates of obesity. This study investigated the views of own body weight and perceived risk of developing NCDs in South African adults. This was a community-based quantitative study. Descriptive statistics were used, and logistic regression analysis was conducted on the data. A total of 1050 respondents took part in the study. Of the 161 respondents who perceived themselves to be normal weight, 98.8% (*n* = 159) misperceived their body weight. The majority of respondents (>90%) who were overweight according to the calculated BMI perceived no risk of developing obesity-related diseases. Most of the respondents, 46% (*n* = 253), believed that body weight was influenced by heredity. The method used for weight loss by 57% (*n* = 173) of the respondents was exercising at home. There was a statistically significant association between Body Mass Index (BMI), employment status, risk of developing diabetes, and body weight misperception (*p* < 0.05). Misperception of body weight was common among the study respondents and may influence weight control intervention strategies. Health promotion targeting personal behaviour, such as body weight self-perception, is crucial in supporting targeted strategies to address obesity in South Africa.

## 1. Introduction

Obesity has become the most serious public health problem worldwide and in South Africa and poses a substantial challenge in preventing non-communicable diseases. In 2016, the global prevalence of obesity among adults was 650 million [1]. An equivalent of 71% of all deaths globally was attributed to non-communicable diseases (NCDs), of which obesity is a risk factor [2]. South Africa ranked first in having the highest obesity rates (28.3%) among adults in Sub-Saharan African (SSA) in 2016 [3], and non-communicable diseases accounted for 51% of all deaths in the country [4]. The burden of comorbidities, especially NCDs associated with obesity, can harm individuals and society as a whole [5]. In many countries in SSA, obesity is reportedly the most leading risk factor in developing comorbidities such as hypertension and diabetes in adults [6]. The dietary shift from consuming traditional foods to a Western diet, such as increased fatty foods and fast food consumption, is instrumental in increasing obesity rates [7].

In addition to the dietary shift, personal behaviour, such as misperception of one’s own body weight, has emerged as a vital factor driving overweight and obesity [8]. Underestimation of one’s own body weight is more likely to be reported by individuals who are overweight and obese irrespective of their age [9]. For example, underestimation of body weight, which was observed among African-Americans who had higher Body Mass Index (BMI) and considered themselves to be overweight, can subsequently lead to the overall underestimation of obesity rates in their population [10]. It has also been argued that the misperception of body weight across the body mass index spectrum, whether normal, overweight, or obese, could be associated with biological indicators of poor health [11]. Other authors claim that underestimating weight status by individuals who are overweight or obese can be beneficial, as they would gain less weight over time [12]. This view, however, has not been proven beyond doubt.

Some researchers in Malaysia [13] and Ghana [14] have reported in their studies that women who were clinically diagnosed as obese, having diabetes mellitus and hypertension, acknowledged that being overweight or obese influenced their acquisition of these chronic diseases. Furthermore, there is a view, based on longitudinal cohort studies done in the U.S. and the U.K., that individuals who perceive themselves as overweight are at increased risk of subsequent weight gain regardless of their actual weight status [15]. Contrary to these views, some individuals who are obese may not necessarily believe that they are at risk of developing these disease conditions [16]. Conversely, the acceptability of larger body weight as ideal and its association with “dignity, respect, health, wealth and strength” are what precipitates weight gain, especially in African countries [17]. However, no research reports this behaviour and notion in South Africa, where obesity remains a serious challenge and a threat to public health.

Thus, there are gaps in the knowledge on body weight self-perception, obesity risks to the acquisition of NCDs, and weight management behaviour in the South African context. Given the impact of body weight perception on the development of NCDs and weight control behaviour, this study aimed to investigate the self-perception of one’s own body weight and the risk of developing NCDs in South African adults.

## 2. Materials and Methods

### 2.1. Research Design

This was a community-based cross-sectional quantitative study to describe the self-perception of one’s own body weight and the risk of developing NCDs. The sample comprised adults 18 years of age and older recruited from urban and rural areas of three provinces in South Africa (Gauteng, North West, and Mpumalanga). Sampling from different settings in the three provinces increased the representation of the various ethnic groups in the study. It is recommended in principle that a sample should be as large as possible in a quantitative study to minimize potential error [18]. To meet this standard, the sample size was calculated using the following formula based on the method described by Cochran [19] given an unknown population size:

*n* = Z^2^·pq/e^2^ (1)

where Z = confidence level (1.96) for 95% level of confidence; p = the estimated proportion of the population; q = 1 − p (reduced to 0.3) to obtain a larger sample size; and e = margin of error (confidence interval). The more heterogeneous the population, the larger the sample size required to obtain a given level of precision.

The sample size was calculated based on a 95% confidence level, 0.5 standard deviations, and a margin of error of 2%. This brought the sample size to 1441.

### 2.2. Data Collection

Data were collected over a period of three months, from March to June 2016 by the lead author and field workers who were trained in the objectives of the study, completion of the questionnaire, anthropometric measurement, and recruitment of study respondents prior to data collection. A systematic random sampling strategy was used to select the respondents. The researchers set out gazebo tents in shopping malls and in other community halls or centres used for community activities to conduct data collection. After selecting the first individual, every third person who passed the tents was selected to form part of the sample. Respondents were approached individually, information about the study was read to the respondents, and those who voluntarily agreed to participate in the study signed an informed consent form.

The structured interviewer-administered questionnaire used to collect data for this study was adapted from a similar study [14]. The questionnaire was modified to obtain information on the socio-demographic characteristics of the respondents, body weight perception, influences of body weight, risk of developing chronic disease conditions, and weight control behaviours. The Cronbach-alpha coefficient (0.77) was calculated from eight items and indicated a high level of reliability of the data collection tool [20].

Measurements of the weights and heights of respondents were taken before the primary interviews in the tents to assure privacy. Weight was measured on a calibrated electronic scale (SECA) to the nearest 0.1 kg with minimal clothing and no shoes. Height was measured using a height measuring tape to the nearest 0.1 cm with no shoes. Body Mass Index (BMI ((kg/m^2^) was calculated using weight and height measurements and was categorized according to the World Health Organization (WHO) classification (underweight < 18.5 kg/m^2^; normal weight = 18.5–24.9 kg/m^2^; overweight = 25.0–29.9 kg/m^2^, and obesity > 30 kg/m^2^ [21]) and was recorded on the questionnaire.

### 2.3. Data Analysis 

Data were coded and captured in a Microsoft Excel spreadsheet, and statistical analyses were performed using STATA version 13 (StataCorp, LLC, College Station, TX, USA), a statistical package for data analysis. Descriptive statistics were analysed, and logistic regression was conducted to identify variables associated with body weight misperceptions. *p*-value less than 0.05 was considered statistically significant.

### 2.4. Ethical Consideration

All the respondents gave their informed consent for inclusion before they participated in the study. The study was conducted per the Declaration of Helsinki, and the protocol was approved by the Medunsa Research Ethics Committee (MREC/H/269/2012: PG).

## 3. Results

### 3.1. Introduction and Socio-Demographics

The study sample that consented and completed the questionnaires was comprised of 1050 respondents. The majority of these respondents (67%, *n* = 711) were aged between 18 and 35 years, 56% (*n* = 586) were females, 70% (*n* = 713) were single, 26% (*n* = 268) had secondary education, and 53% (*n* = 547) were employed. Sixty-four per cent (*n* = 668) resided in an urban area. A total of 424 (40%) respondents was of normal weight, 28% (*n* = 290) were overweight, and 26% (*n* = 272) were obese (Table 1).

### 3.2. Perceived Bodyweight

Reflected in Table 2 are respondents’ own perceived body weights compared to the calculated BMIs. All the respondents who perceived themselves to be underweight (*n* = 5) misperceived their body weight. Of the respondents (*n* = 161) who perceived themselves to be normal weight, 98.8% (*n* = 159) misperceived their body weight according to the calculated BMI. Among the respondents who were overweight and obese, (*n* = 381) misperceived their body weight.

### 3.3. Perceived Risk of Developing NCDs

When the respondents were asked about their perceived risk of developing NCDs, of those who were not overweight according to calculated BMI, 8% (*n* = 19) responded that they were not at risk of developing high blood pressure (HBP), 7% (*n* = 25) responded they were not at risk of developing diabetes mellitus (DM), and 7% (*n* = 21) responded they were not at risk of developing CVDs. For those who were overweight according to calculated BMI, 95% (*n* = 331) responded that they were at risk of developing HBP, 95% (*n* = 231) responded they were at risk of developing DM, and 95% (*n* = 295) responded that they were at risk of developing CVDs. The majority of respondents (>90%) who were overweight according to the calculated BMI responded that they were not at risk of developing HBP, DM, or CVDs (Table 3).

### 3.4. Perceived Factors Influencing Body Weight

**Figure 1:**
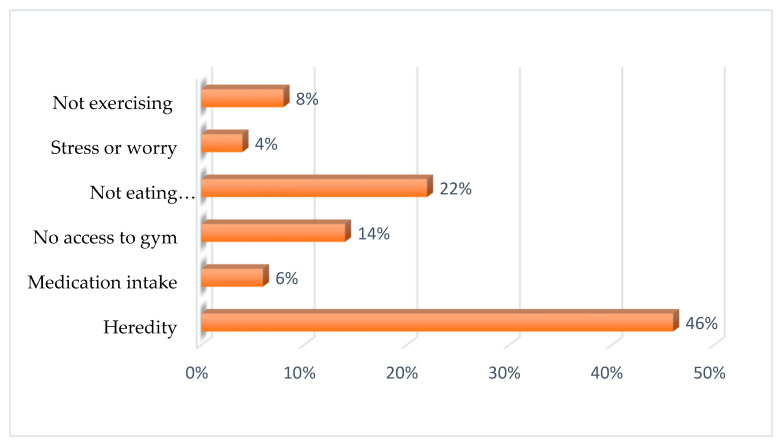
Perceived factors that influence body weight according to the respondents.

To understand the influencers of body weight, respondents were asked what they believed influences body weight. Respondents believed that heredity factors (46%, *n* = 253), not eating healthy food (22%, *n* = 122), having no access to the gym (14%, *n* = 80), and not exercising (8%, (*n* = 43) influence body weight (Figure 1).

### 3.5. Self-Reported Weight Management Practices

To understand the respondents’ views on weight management, they were asked about weight loss attempts; 58% (*n* = 319) said they tried losing weight, and 42% (*n* = 230) did not attempt to lose weight.

**Figure 2:**
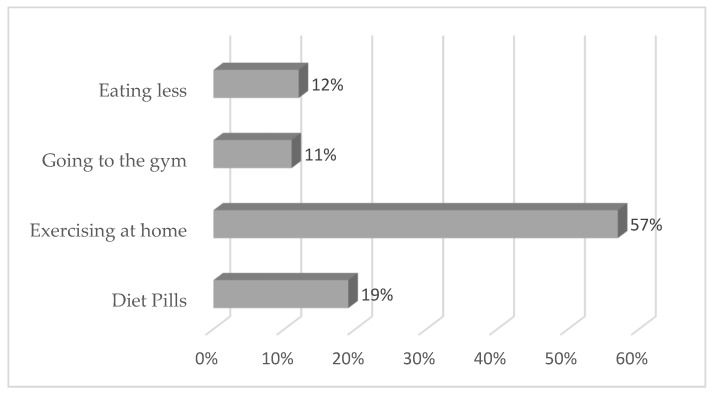
Reported methods used for weight loss.

Those who tried to lose weight were asked about the methods they used to lose weight; over half, 57% (*n* = 173) said they exercised at home, 19% (*n* = 57) used diet pills, and 12% (*n* = 37) said they ate less (Figure 2).

**Figure 3:**
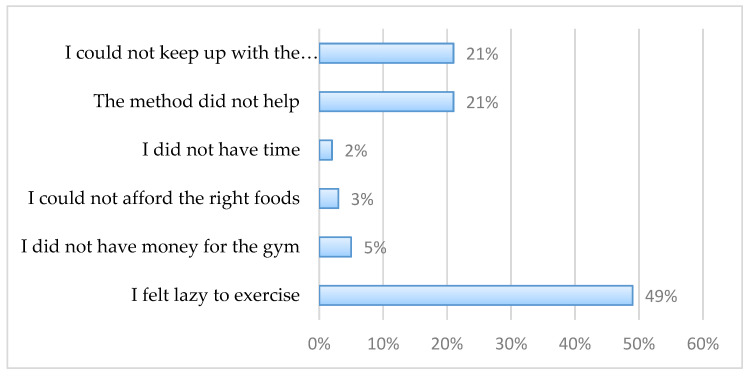
Reasons for failed attempt to lose weight.

Respondents who tried losing weight and failed provided their reasons for failure to lose weight. Almost half, 49% (*n* = 73), cited being too lazy to exercise, 21% (*n* = 31) stated they were unable to keep up with the programme, and 21% (*n* = 31) said the method used did not help (Figure 3).

**Figure 4:**
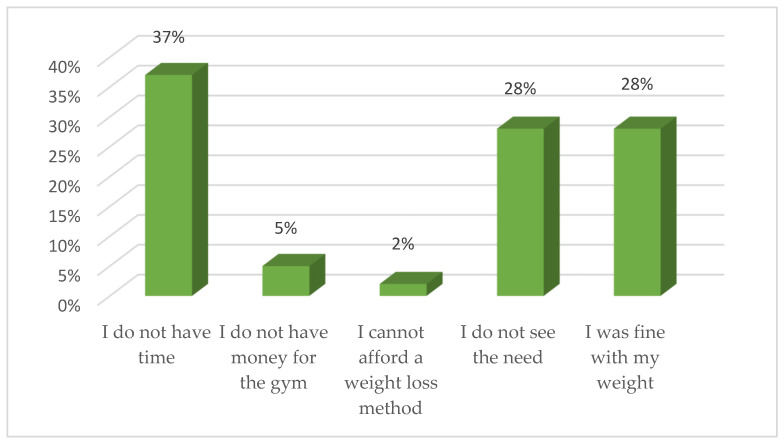
Reasons for not trying to lose weight.

Of the 230 respondents who did not try to lose weight, 37% (*n* = 91) said they did not have time, 27.6% (*n* = 68) did not see the need to lose weight, and 28% (*n* = 70) said they were fine with their weight (Figure 4).

### 3.6. Association between Misperception of Body Weight and Variables

A bivariate and a multivariate logistic regression analysis were conducted to assess the association of the independent variables and misperception of body weight. We found an association between BMI, employment status, risk of developing HBP, DM, high cholesterol, and CVDs and misperception of body weight. The association was statistically significant (*p* values < 0.05) in the bivariate analysis. The variables that had a *p* value < 0.05 in the bivariate analysis were used in the multivariate model. The BMI, employment status, and the risk of developing diabetes mellitus remained statistically significantly associated with misperception of body weight (*p* < 0.05) in the multivariate analysis (Table 4).

## 4. Discussion

This cross-sectional study aimed to investigate the self-perception of body weight and the risk of developing NCDs in South African adults and offers insight into body weight perceptions and the risk of developing NCDs.

The study findings showed misperception of body weight, with the majority of individuals perceiving themselves to be of normal weight, whereas they were overweight according to the calculated BMI. These findings are in line with what has been reported in a previous South African study [22]. Similarly, in a study conducted in the U.S., those who were overweight and obese underestimated their body weight [23]. The misperception of body weight status observed among the respondents in the study was associated with their BMI by a factor of 0.94 (95% CI (0.91–0.98), *p* < 0.05). Even though the majority of the respondents in the study was female, there was no association between sex and body weight misperceptions. These findings are contrary to the findings of the authors of [13,14], who reported that women were more likely to misperceive their body weight status. Undoubtedly, the underestimation of weight status is a probable health threat that can prevent much needed behavioural change in order to improve health. However, some authors equate the mismatch between perceived weight and BMI status as representing a lack of awareness of "healthy" weight among individuals [24]. Understanding the concepts of normal weight, overweight, and obese is crucial as it affects the perceptions of weight and its relation to obesity-related diseases, even amongst those who are not obese.

In contrast, men and women who were obese (BMI ≥ 35) in the U.S. had the most accurate body weight perception [25]. However, exposure to obesity is likely to influence body weight perception towards making larger body weight more acceptable. This is the case in most African studies where larger body weight is reportedly valued and perceived as a sign of affluence [17]. In South Africa, fatness is still perceived to be a symbol of “beauty, confidence, happiness, wealth, and affluence” [22]. These beliefs harm the perception of body weight, and individuals may ignore measures to address obesity.

In the current study, even though more than 90% of the respondents who were overweight and obese according to the calculated BMI perceived they were at risk of developing cardiovascular diseases, diabetes, and high cholesterol, more than 90% still perceived that they were not at risk. The findings further showed that the risk of developing disease conditions such as diabetes was associated with the misperception of body weight status by a factor of 0.61 (95% CI 0.37–1.00), *p* = 0.05). However, the findings were based on reported perceived risk, and it was not confirmed in the study if individuals had ever been diagnosed with any obesity-related disease conditions. Researchers in a study conducted in Cape Town [8] reported that overweight women who had diabetes and hypertension had perceived obesity as a risk for these disease conditions. Conversely, some authors believed that, even though a chronic disease is seen as the strongest predictor of the perception of health, overweight individuals may believe that they are at a lower risk [26]. Such beliefs have negative public health implications, as individuals may not correctly know the dangers of acquiring NCDs. The rate of NCDs related to obesity continues to rise, and Southern Sub-Saharan Africa has the highest rates of diabetes and cardiovascular disease, which become costly to the health care system of a country [27]. Understanding the health risks associated with overweight and obesity will enable individuals to implement strategies to prevent related health problems.

The study findings showed that the majority of the respondents believed that body weight is influenced by heredity. Bosire et al. [22] reported similar findings in which obesity was attributed to heredity, and therefore individuals felt they had no control over it. On the other hand, the authors of [14] believed that overweight individuals themselves believe that heredity influences obesity. Therefore, individuals can argue that being obese is part of their families and, therefore, their body weight is considered acceptable. The impact of these beliefs is that, even though they are not related to weight itself, they strongly influence an individual's body weight perception. Thus, for public health education, campaigns, and intervention strategies to effectively combat overweight and obesity, efforts should also be targeted at addressing individuals' beliefs about body weight influences.

The majority of individuals in the study reported that they had tried to lose weight, primarily by exercising at home. A systematic review confirms that individuals who perceived themselves as overweight were more likely to attempt to lose weight [28]. A study conducted in the U.S. argues that women might engage in weight-loss strategies to improve their appearance [29]. Similar views are held by researchers in a study conducted in Nigeria [30], where the authors found that engagement in weight loss programmes among young women was motivated by a desire to look good rather than by health concerns. However, in the current study, exercising at home might have been misreported by the respondents, as most perceived that they were of normal weight.

In essence, not all methods that seem to produce results in other countries or even in developing countries may be advocated for the South African adult population without understanding their body weight perceptions and weight management behaviours. Weight management is a fundamental public health behaviour change strategy to address the increasing rate of obesity. As a strategy, weight loss is vital for the management of overweight and obesity, wherein a weight loss of 5 to 10% is likely to produce health benefits and improvements in blood pressure, blood cholesterol, and blood sugar levels [31].

The results of the logistic regression of 1.61 (95% CI 1.12–2.31), *p* < 0.05) show an association between employment status and misperception of body weight. The greatest challenge could be that, even though the majority of the respondents are employed, they reside in townships where they are exposed to high energy, dense, high fat, high sugar foods, and fast foods [32,33]. These foods are cheaper and easily available and make individuals susceptible to weight gain. Thus, the shift from traditional foods is worrying for public health planners, as the consumption of food items high in energy and fat contributes to an increase in overweight and obesity. Public health interventions and strategies should address the food environment in communities to combat overweight and obesity.

In the current study, the individuals who attempted to lose weight and failed attributed this to being too lazy to exercise. Stigma and stereotypes against people who are overweight cannot be ignored, as they influence their motivation to engage in weight management strategies to improve their health. Such stereotypes, for example, that obese people are “lazy, lack self-discipline, and have poor willpower,” exist and can influence engagement in weight management strategies, either positively or negatively [34]. Moreover, a study conducted in Norway revealed that overweight individuals might be sensitive about their body weight and may be reluctant to engage in weight management behaviours [35]. In general, lack of weight management participation can have adverse public health implications, and therefore the challenge for public health is to engage the relevant stakeholders to communicate health benefits that result from weight management.

### Study Limitations

The findings of this study must be understood in the context of certain limitations. The study was conducted among diverse populations from different ethnic groups and geographical areas, and the findings cannot be generalized to the larger population in terms of context, setting, and respondents. The second limitation is that the study was conducted at a time when most people were at their places of work. Therefore, future studies should address these limitations.

## 5. Conclusions

There was a misperception of body weight among the study respondents. The risk of developing NCDs was seen as likely by those who were overweight and obese. Therefore, those who are normal weight might downplay their risk of developing NCDs. Correct perception of body weight may therefore influence the success of tailor-made, effective education and behaviour intervention strategies for overweight and normal-weight adults, leading to healthy behaviour in society as a whole. With the increased prevalence of obesity among South African adults, these findings are crucial to informing effective public health intervention strategies. Future studies on the current topic are therefore recommended to establish the extent of body weight misperceptions among the adult population.

## Figures and Tables

**Table 1:** Sociodemographic characteristics of the respondents (*n* = 1050).

Socio-Demographic Characteristics	*n*	%
Age		
18–35 years	711	67
36–55 years	280	27
Older than 55 years	58	6
Sex		
Females	586	56
Males	463	44
Marital status		
Ever married	311	30
Single	713	70
Educational level		
No formal education	11	1
Primary education	48	4
Secondary education	268	26
Tertiary education	258	25
Employment status		
Not employed	494	47
Employed	547	53
Area of residence		
Urban	668	64
Rural	373	36
BMI classification		
Underweight (<18.5 kg/m^2^)	64	6
Normal weight (18.5–24.9 kg/m^2^)	424	40
Overweight (25.0–29.9 kg/m^2^)	290	28
Obese (30.0 kg/m^2^ and above)	272	26

**Table 2:** Respondents’ perceived body weight and calculated BMI.

Perceived Weight Status	Correctly Perceived Weight		Misperceived Weight		Total
	*n*	%	*n*	%	
Underweight (<18.5 kg/m^2^)	0	0	5	100.0	5
Normal weight (18.5–24.9 kg/m^2^)	2	1.2	159	98.8	161
Overweight and obese (25.0–above 40 kg/m^2^)	0	0.0	381	100.0	381

**Table 3:** Respondents’ perceptions of risk of developing NCDs and the calculated BMIs.

Perception of Risk of Developing NCDs	Not Overweight		Overweight	
	*n*	%	*n*	%
High blood pressure				
No	19	8	224	92
Yes	17	5	331	95
Diabetes mellitus				
No	25	7	323	93
Yes	11	5	231	95
High cholesterol				
No	24	8	296	93
Yes	12	4	257	96
CVD				
No	21	7	260	93
Yes	15	5	295	95

Not overweight BMI < 25.0 kg/m^2^; overweight BMI > 25.0 kg/m^2^.

**Table 4:** Logistic regression examining factors associated with misperception of body weight.

Variables	Misperception of Body Weight (Bivariate Analysis)			Misperception of Body Weight (Multivariate Analysis)		
	Odds Ratio	95% CI	*p* Value	Odds Ratio	95% CI	*p* Value
Age	0.98	0.97–1.00	0.09	-	-	-
Gender	0.88	0.61–1.25	0.48	-	-	-
Body Mass Index	0.93	0.90–0.97	* 0.00	0.94	0.91–0.98	* 0.00
Education level	1.17	0.97–1.41	0.08	-	-	-
Employment status	1.59	1.13–2.24	* 0.00	1.61	1.12–2.31	* 0.00
Marital status	1.18	0.84–1.67	0.32	-	-	-
Area of residence	0.98	0.68–1.39	0.92	-	-	-
Weight management behaviour	1.27	0.97–1.66	0.07	-	-	-
Risk of developing high blood pressure	0.54	0.38–0.76	* 0.00	-	-	-
Risk of developing diabetes mellitus	0.45	0.32–0.64	* 0.00	0.61	0.37–1.00	* 0.05
Risk of developing high cholesterol	0.49	0.35–0.69	* 0.00	-	-	-
Risk of developing cardiovascular diseases	0.51	0.36–0.72	* 0.00	-	-	-

CI—confidence interval; * *p* value statistically significant.

## Data Availability

The data presented in this study are available upon request from the corresponding author.
